# Eco-friendly and biocompatible gelatin plasmonic filters for UV-vis-NIR light

**DOI:** 10.1038/s42004-024-01202-6

**Published:** 2024-05-25

**Authors:** I. Brian Becerril-Castro, Yoel Negrín-Montecelo, Josep Moreno, Miguel A. Correa-Duarte, Vincenzo Giannini, Ramón A. Alvarez-Puebla

**Affiliations:** 1 Department of Physical and Inorganic Chemistry, Universitat Rovira i Virgili, Carrer de Marcel∙lí Domingo 2-4-6, 43007 Tarragona, Spain; 2 Deliranto, Carrer de Llevant, 7, 43840 Salou, Spain; 3 CINBIO, Universidade de Vigo, 36310 Vigo, Spain; 4 Instituto de Estructura de la Materia (IEM), Consejo Superior de Investigaciones Científicas (CSIC), Serrano 121, 28006 Madrid, Spain; 5 Technology Innovation Institute, Masdar City, Abu Dhabi United Arab Emirates; 6 Centre of Excellence ENSEMBLE3 sp. z o.o., Wolczynska 133, Warsaw, 01-919 Poland; 7 ICREA, Passeig Lluís Companys 23, 08010 Barcelona, Spain

**Keywords:** Organic-inorganic nanostructures, Nanophotonics and plasmonics

## Abstract

The quest for environmentally sustainable materials spans many fields and applications including optical materials. Here, we present the development of light filters using a gelatin-based nanocomposite. Owing to the plasmonic properties of metallic nanoparticles (NPs), strong light-matter interactions, these filters can be customized across the UV-Visible-NIR spectrum. The filters are designed for modular use, allowing for the addition or removal of desired spectral ranges. Moreover, the nanocomposites are composed of biodegradable and biocompatible materials which highlight the intersection of chemistry and ecological awareness for the exploration of new eco-friendly alternatives. These plasmonic gelatin-based filters block light due to the Localized Surface Plasmon Resonance (LSPR) of the NPs and can be tailored to meet various requirements, akin to a diner selecting options from a menu. This approach is inspired by culinary techniques, and we anticipate it will stimulate further exploration of biomaterials for applications in optics, materials science or electronics.

## Abstract (web summary)

Optical filter materials are used across scientific disciplines for imaging or spectroscopy, but inexpensive and eco-friendly alternatives remain underexplored. Here, the authors exploit the localized surface plasmon resonance of metal nanoparticles embedded in edible gelatine for modular light filters working in the ultraviolet to near-infrared range.

## Introduction

The increasing consumer demand for environmentally friendly products has shifted research efforts toward the development of eco-friendly materials and sustainable processes. Among these new materials, nanocomposites that incorporate biopolymers occupy a prominent position, primarily because biopolymers are naturally occurring in living organisms and are presumed to be biodegradable and biocompatible. Interestingly, the use of biopolymers is also prevalent in the food industry. For instance, plant-based guar gum and bacteria-derived xanthan gum are biopolymers commonly used as thickening and stabilizing agents^1–3^. However, gelatin holds a unique place among the biopolymers used in cooking. Gelatin is a protein-based substance derived through the hydrolysis of animal connective tissues. It is highly soluble in water and is also considered nutritious due to its high protein content (85–92%), containing all essential amino acids except for tryptophan, and its low caloric value^4^. Additionally, its unique melt-in-the-mouth property, due to the formation of thermally reversible gels with a gel-melting point below body temperature (<35 °C), is highly prized for making gel desserts^5^. As it is edible, gelatin capsules and microcapsules are commonly found in pharmaceuticals^4^.

Nanocomposites with gelatin include works with carbon nanotubes^6^ and copper nanoparticles (NPs)^7^ to create conductive materials. Nonetheless, the development of gelatin-based nanocomposites for photonics and optics has not been extensively studied^8^. In this work, we explore an intriguing application for optical uses: the design and fabrication of gelatin-based nanocomposites for light filters. To impart optical properties to a traditionally transparent material^9^, we incorporated a variety of metallic NPs into the polymeric matrix, providing mechanical support. Due to their size, metallic NPs can sustain localized surface plasmon resonance (LSPR), enabling light manipulation (entrapment) at the nanoscale. By varying the physical characteristics of the NPs, such as material, size, and geometry, it is possible to finely tune their optical properties across the electromagnetic spectrum. The inclusion of NPs allows us to work within the UV-Visible-NIR range with high control over the optical filtering properties of the nanocomposites. These plasmonic gelatin-based light filters exhibit modular behavior, allowing them to be used in combination to add or remove the desired spectral range of interest, while being composed of sustainable and ecological materials. Our work presents an alternative approach to the production of light filters that meets requirements in a manner comparable to selecting options from a restaurant menu.

## Results and discussion

**Fig. 1:**
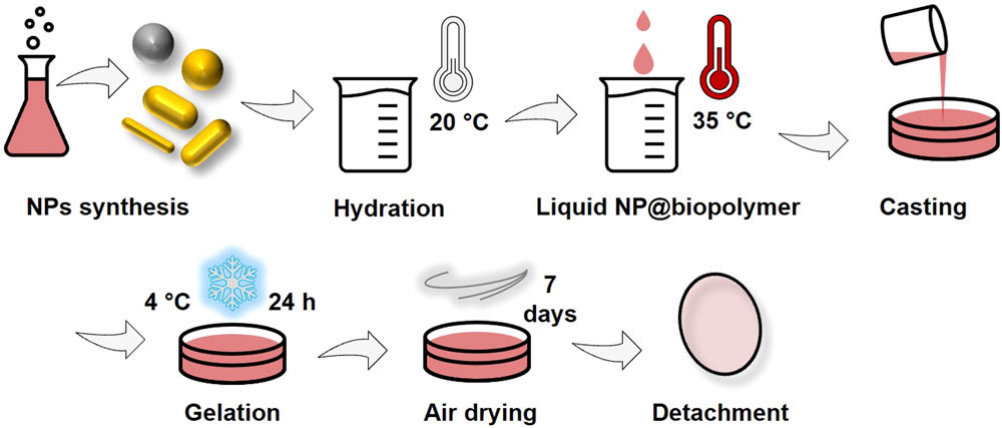
Schematic procedure to obtain plasmonic gelatine-based light filters. Five types of NPs with absorption within ultraviolet to near-infrared range were mixed with pre-dissolved gelatin to create a NP@biopolymer solution. After casting, the molds were refrigerated, exposed to air for drying, and finally extracted.

To create plasmonic gelatin-based light filters, we alter the inherent optical properties of plain gelatin by dispersing plasmonic NPs. Combining the optical transparency of gelatin with the absorption capabilities of metallic NPs, we hypothesized that achieving the desired filtering effect was contingent on preventing NP agglomeration, which could otherwise obscure the distinct SPRs of the NPs. Accordingly, we devised and adhered to the procedure outlined in Fig. 1. Initially, five types of NPs were chosen and synthesized using established methods: silver nanospheres (AgNPs), gold nanospheres (AuNPs), and gold nanorods (AuNRs) with three varying aspect ratios (ARs). Subsequently, these NPs were amalgamated with pre-dissolved gelatin to create an NP@biopolymer solution, which was then cast in Petri dishes. Post-casting, the molds were refrigerated at 4 °C for 48 hours to ensure solidification (gelation). Once set, the nanocomposites were exposed to air for drying. Finally, the dry material was carefully extracted using tweezers.

**Fig. 2:**
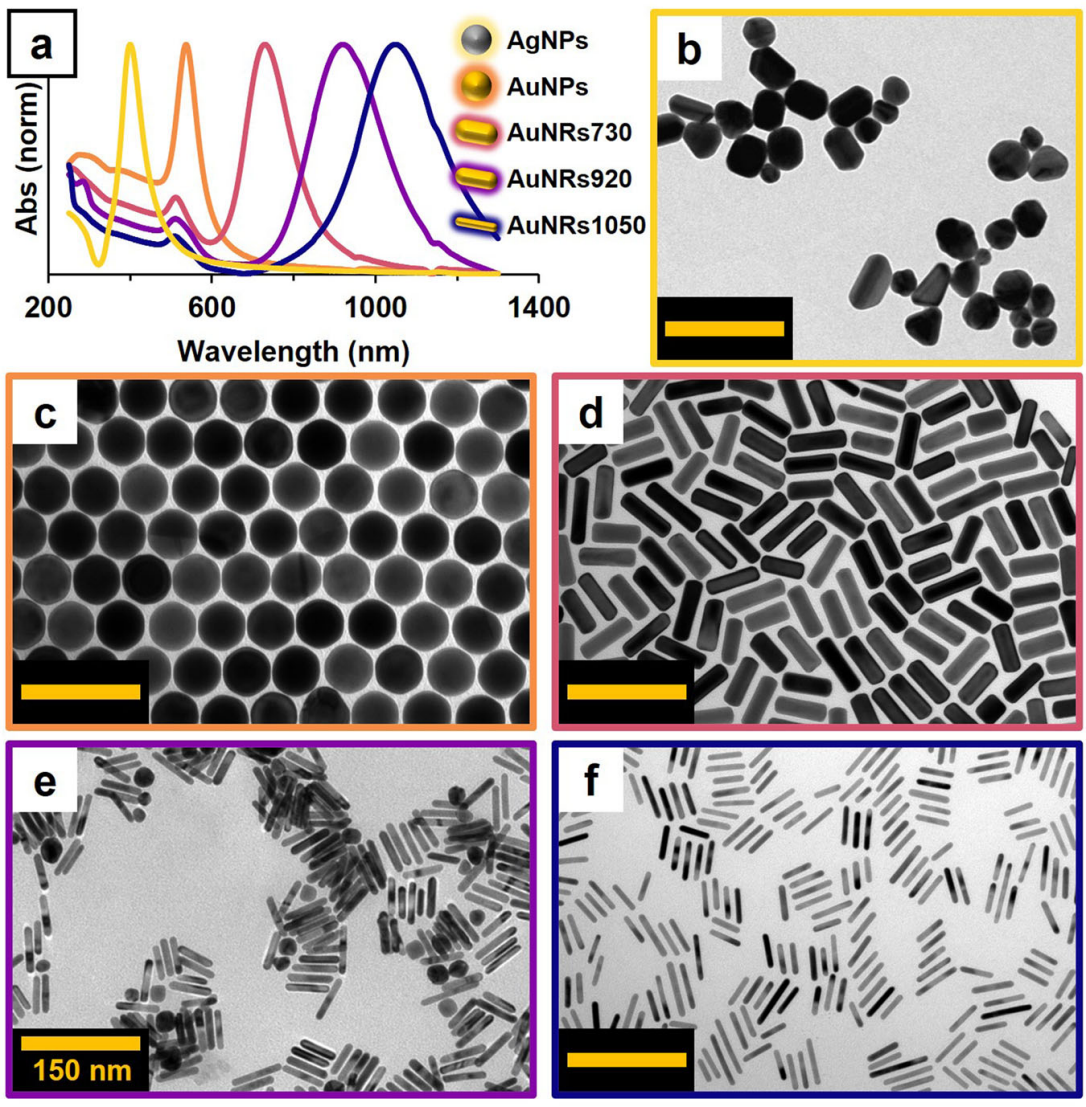
Optical and electronic characterization of the metallic NPs employed in the fabrication of plasmonic gelatine-based light filters. **a** Experimental surface plasmon resonances of the NPs obtained by UV-vis spectroscopy. From left to right: AgNPs (405 nm), AuNPs (537 nm), AuNRs (730, 920 and 1050 nm). **b**–**f** Representative TEM images for AgNSp (Φ = 51 nm), AuNPs (Φ = 61 nm), AuNRs730 (AR = 3.1), AuNRs920 (AR = 5.2) and AuNRs920 (AR = 6.5).

The chosen set of NPs demonstrated robust absorption across the UV-Vis-NIR spectrum due to LSPR, as evidenced by the absorption spectra in Fig. 2a. Plasmon resonances were identified at 405 nm for AgNPs, 537 nm for AuNPs, and at 730, 920, and 1050 nm for AuNRs. This extensive absorption range underscores the ability to adjust the optical properties of NPs. Representative TEM images for each NP type are displayed in Fig. 2b–f. The average sizes were 51 ± 13 nm for AgNPs and 61 ± 2 nm for AuNPs. Meanwhile, the lengths of AuNRs were 69 ± 6, 51 ± 4, and 50 ± 12 nm, correlating with increasing AR of 3.1, 5.2, and 6.5 for AuNRs730, AuNRs920, and AuNRs1050, respectively. Notably, while all NPs had comparable sizes, their geometry and composition markedly influenced their optical responses. Specifically, the AR of AuNRs facilitated controlled responses in the Vis-NIR region^10^, while the AgNPs’ LSPR bordered the visible and UVA regions^11^.

Direct addition of dry biopolymer (laminates) to the NP colloid resulted in NP aggregation. Therefore, the biopolymer was first hydrated (10 min in cold water), then dissolved (at 35 °C), and finally combined with a predetermined volume of NPs. To calculate the necessary NP volume (${v}_{{np}}$) with absorbance ${Ab}{s}_{{np}}$ (measured in a cuvette with 1 cm light path), we assumed a linear relationship between the NPs’ absorption and the optical path length. Using a standard petri dish as a mold (diameter = 9 cm) and targeting a final concentration of 2% biopolymer in a 15 ml volume for an easily detachable nanocomposite, we employed the expression:

1 $${v}_{{np}}={\pi 4.5}^{2}\frac{{\log }_{10}\left(\frac{100 \% }{100 \% -A\left( \% \right)}\right)}{{ab}{s}_{{np}}},$$

where A is the desired maximum absorption (derivation in Supplementary Note 1). After determining the NP volume, water was added to achieve a final 9 ml volume, which was then mixed with a 6 ml aliquot of 5% gelatin. The resultant liquid NP@biopolymer solution was cast in the mold and refrigerated to form hydrated gelatinized nanocomposites. Any present bubbles were removed while the mixture was still liquid to ensure material uniformity.

**Fig. 3:**
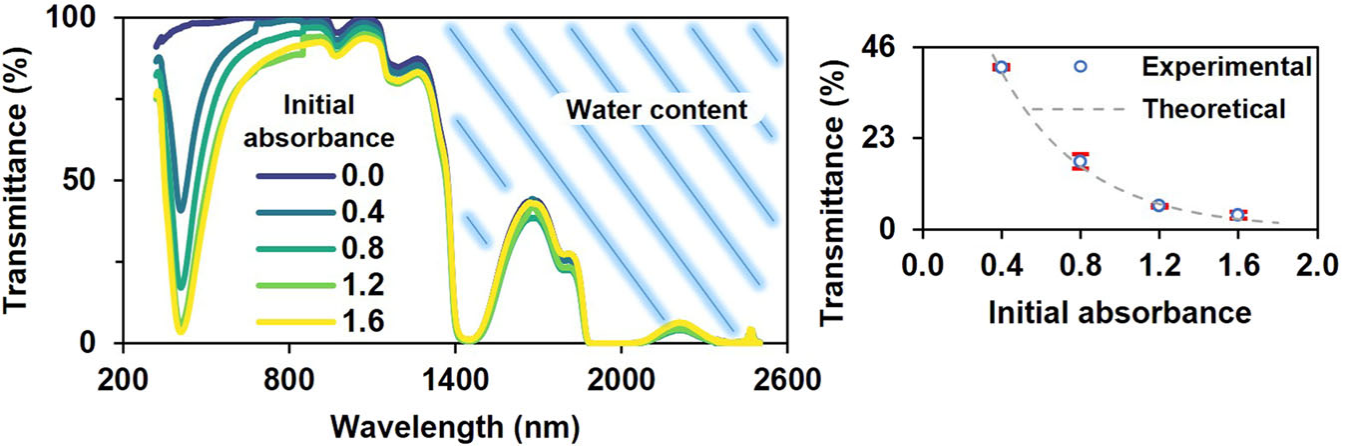
UV-Vis-NIR characterization of the hydrated nanocomposite. The absorption from 1320 nm onward is likely due to the material’s water content. Inset: measured transmittance (dots) of the estimated absorbance. Error bars represent standard deviation. Theoretical result as gray dashed line. Measurements as blue circles. Absorbance 0 correspond to the hydrated biopolymer without AgNPs.

We examined the formation of hydrated gelatinized nanocomposites by preparing and analyzing five samples with varying concentrations of AgNPs using UV-Vis-NIR spectroscopy (Fig. 3). A pronounced absorption at 410 nm, attributed to the AgNPs, was observed. The 5 nm redshift of the absorption band aligns with the expected change in the dielectric constant due to the biopolymer. Absorption from 1320 nm onward was attributed to the material’s water content. Correlating the absorbance of the liquid (0.4, 0.8, 1.2, and 1.6) before gelation with the experimental transmittance of the hydrated NP@biopolymer (at 410 nm) indicated good agreement with the predicted transmittance, suggesting that the gelation process does not cause NP aggregation but instead immobilizes their position within the three-dimensional biopolymer matrix. This result also agrees with works that propose that the positively charged gelatin can be electrostatically adsorbed on negatively charged metal NPs to form stable gelatin-coated NPs^12,13^.

**Fig. 4:**
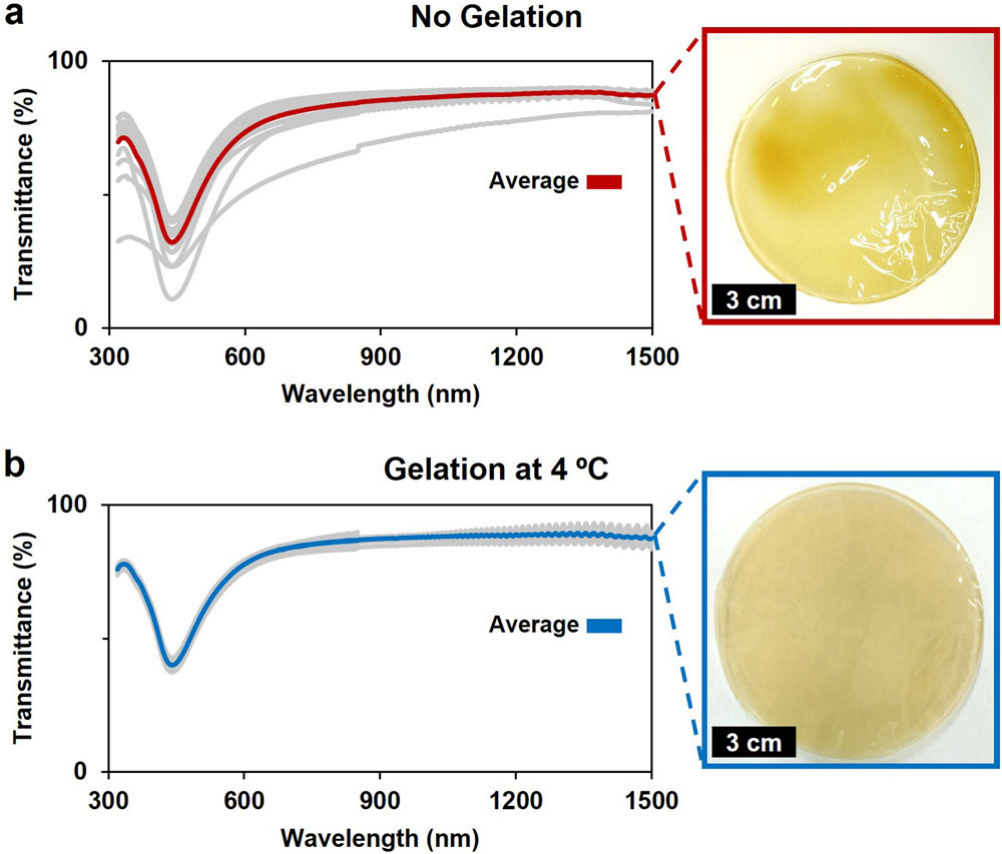
Impact of the gelation process in the homogeneity of the filters. UV-Vis-NIR spectra of **a** filters obtained without and **b** with gelation, average in red and blue, respectively. The transmittance of 15 random points distributed on the surface of each material is depicted as gray lines. As inset, the corresponding optical image.

Notably, refrigeration was essential for the gelation of the NP@biopolymer, distinct from pure gelatin. Without this step, the final filters were inhomogeneous. Figure 4 shows the effect of the gelation process on the homogeneity of the filters. Without gelation (Fig. 4a) an inhomogeneous material is obtained, characterized by a broad distribution in the transmittance and therefore NPs is present in the sample. With gelation (Fig. 4b), the light filters are homogeneous and characterized by a small dispersion in the transmittance. This result can be understood because as the solution cools, the gelatin molecules start to bond together to form a solid network. This network traps water molecules, coated and free NPs within its structure. Without this process, the evaporated water causes the formation of clusters with high concentration of particles, as seen in the optical images. Additional filters that were not refrigerated with increasing Ag concentration are shown in Supplementary Fig. 1.

**Fig. 5:**
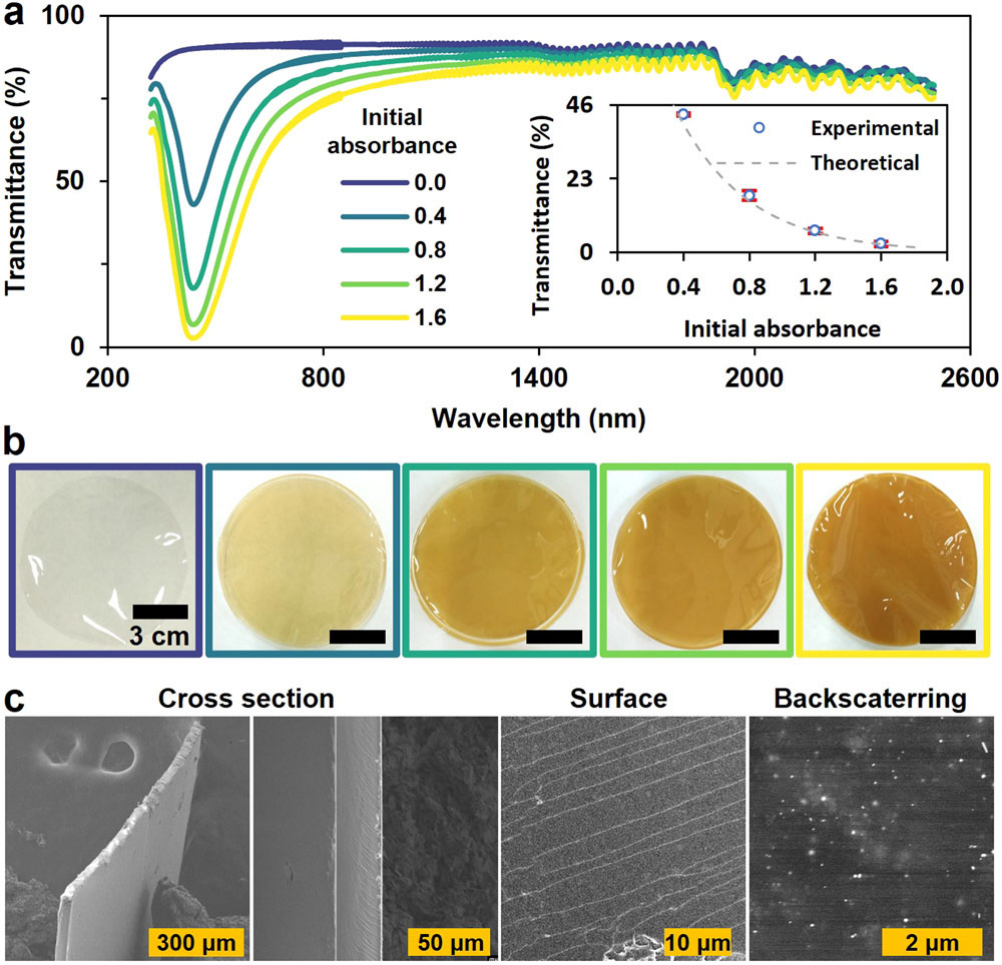
Optical and scanning electronic microscopy characterization of gelatin-based light filters with AgNPs. **a** UV-Vis-NIR spectra. Inset: measured transmittance (dots) of the estimated absorbance. Error bars represent standard deviation. Theoretical result as gray dashed line. Measurements as blue circles. **b** Optical images. **c** SEM images of the cross-section and surface of the filters with secondary electrons. Backscattering image showing the distribution of AgNPs inside the material.

The subsequent drying process aimed to eliminate water’s impact on the nanocomposite’s transmittance and enhance its mechanical properties. For instance, we test that the air-dried plasmonic filters can be used at high temperatures (50° for 3 days) and can also be left unattended under illumination without changes in their optical properties (Supplementary Fig. 2). Figure 5a illustrates that the absorption starting from 1320 nm, visible in Fig. 3, is nearly removed in all samples post-drying (further drying in oven (50°) reduces even more the water content). Although the final transmittance still aligns with theoretical predictions, a broadening of the absorption peak and a decrease in maximum transmittance (89%) are observed. These changes suggest that the AgNPs draw closer as the water content diminishes, forming occasional interactions and thus reducing the maximum absorption by the NPs, which is compensated by the biopolymer’s absorption. However, a distinct absorption band due to the NPs remains. The highest absorption occurs between 436 and 438 nm, which is redshifted compared to the hydrated material and the original colloidal particles. This finding aligns with the increased dielectric medium of the material: *n* = 1.536 for pure gelatin at 632.8 nm^9^. The shiny appearance and uniform color of the filters with high absorption (>50%) over a white background are shown in Fig. 5b. The filters are thin, flexible layers but are prone to tearing. SEM characterization (Fig. 5c) revealed uniform thickness (33.4 µm) and a distinctive layer pattern on the surface, likely imprinted by the mold. Backscattered electron imaging confirmed even particle distribution within the material.

**Fig. 6:**
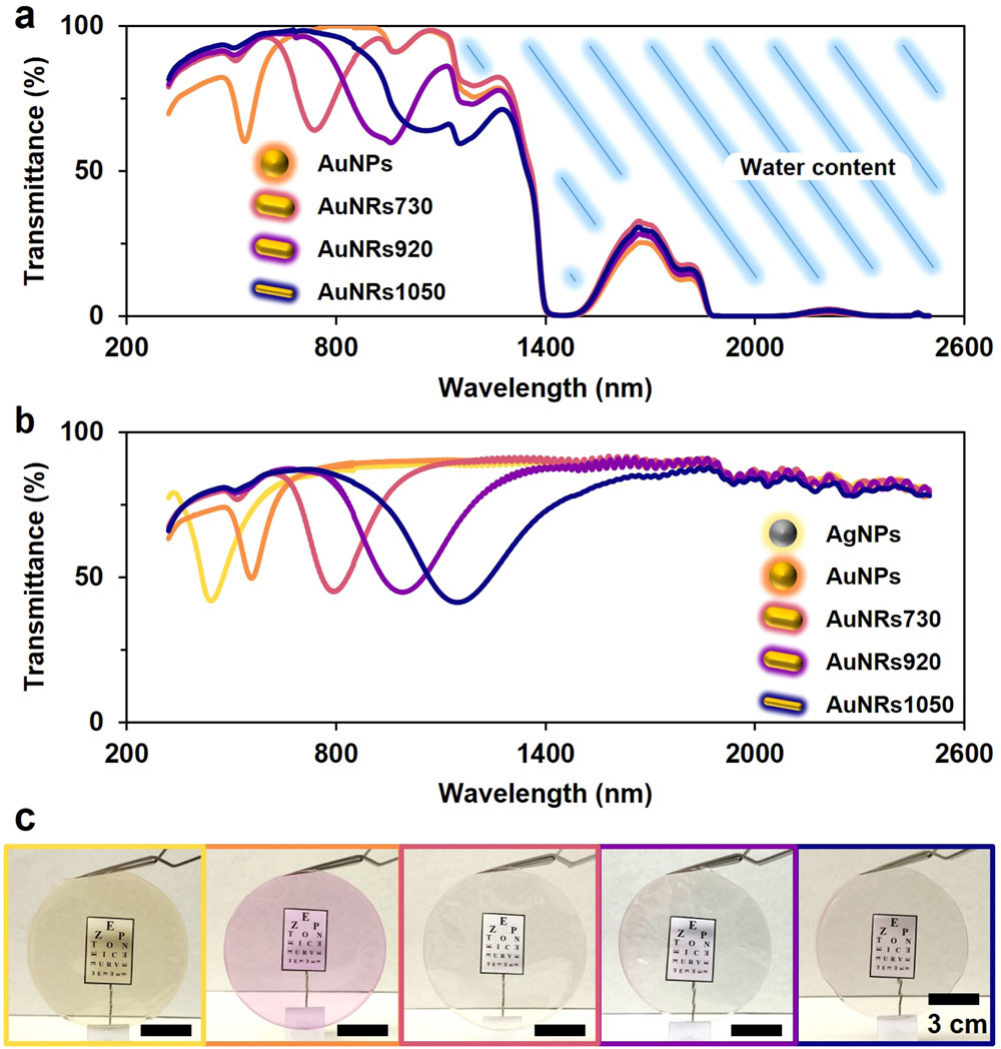
Optical and electronic microscopy characterization of gelatin-based light filters with AgNPs. **a** UV-Vis-NIR spectra of the hydrated material with AuNPs and AuNRs. **b** UV-Vis-NIR spectra of the gelatine-based filters with different NPs. **c** Optical images of the filters in front of a pseudo-tumbling E chart to showcase the visibility of the material.

Having established the methodology for light filters with AgNPs, we extended our process to include all previously selected NPs (i.e., AuNPs, AuNRs730, AuNRs920, and AuNRs1050) for filters with ~60% transmittance. This transmittance level was chosen as a balance between absorption efficiency and ease of preparation. The UV-Vis-NIR spectra of these nanocomposites (Fig. 6a) indicate that the gelation process also prevents aggregation of the AuNPs and AuNRs. However, the drying process differed from that for AgNPs: an increase (~10%) in final absorbance was observed (Fig. 6b). This increase, previously attributed to gelatin, was overshadowed by interparticle interactions in AgNP filters. In AuNP and AuNR filters, this interparticle interaction compensation seems absent. This hypothesis is supported by a smaller broadening of the AuNPs absorption peak compared with the AgNPs. One explanation is that the AuNPs and AuNRs have CTAC and CTAB stabilizing layers, respectively, that attach to the metal surface with an amine group. Thus, compared to the easily displaced citrate on AgNPs, the AuNPs and AuNRs are less bound to the biopolymer during drying and tend to arrange apart from other NPs. The final appearance of the filters is shown in Fig. 6c, alongside a filter with AgNPs for comparison. A small image with letters resembling a “tumbling E” chart is placed behind the filter to demonstrate the material’s transparency. These results show that gelatin-based filters can be tuned within the UV-Vis-NIR spectrum using the employed NPs’ LSPR. Therefore, this procedure can be expanded to other morphologies or materials with a desired absorption spectrum. The gelatin provides a transparent canvas that can be exploited as desired.

**Fig. 7:**
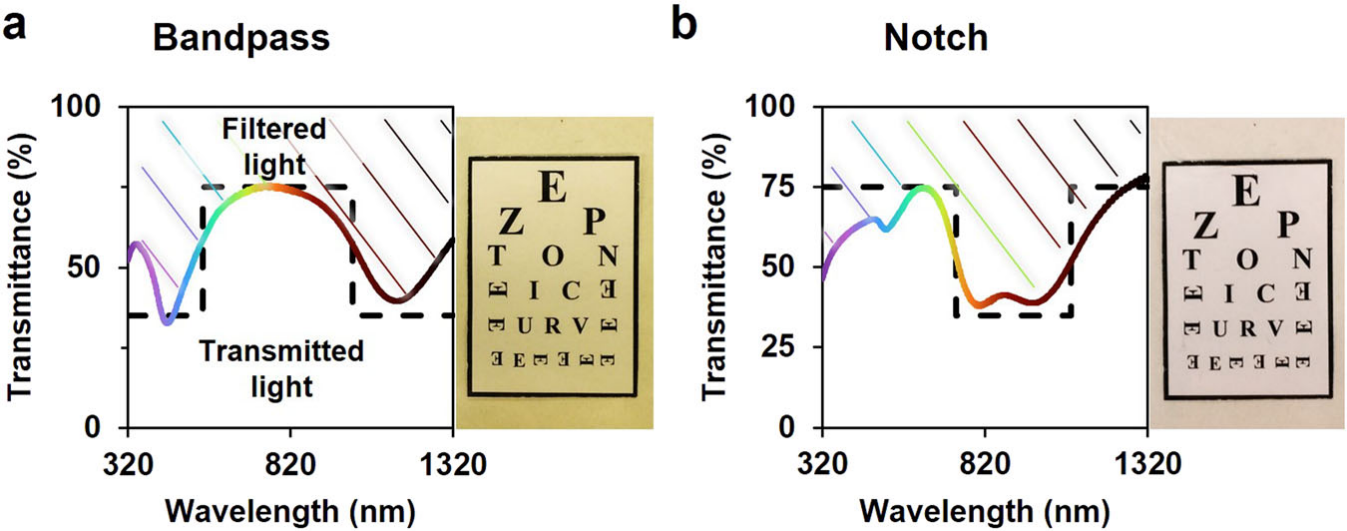
Filter configurations for multiuse of filters. **a** Bandpass configuration in the Vis-NIR range (550–1010 nm) using the filters with AgNPs and AuNRs1050. **b** Notch configuration using the AuNRs730 and AuNRs1050 filters. In dashed lines the ideal approximation.

The process for obtaining biopolymer-based light filters is not limited to selecting constituent NPs but can also be designed through arrangements of filters. This approach is demonstrated using the presented filters to create two commonly used filter configurations: a bandpass filter and a notch filter. In the first example (Fig. 7a), individual AgNPs and AuNRs1050 filters were stacked. This configuration restricts passing wavelengths within the range of 550–1010 nm, effectively acting as a band filter. The optical image over a pseudo-tumbling E chart allows visual inspection of the material, as most of the optical range is permitted to pass through. A different filter arrangement utilizes AuNRs730 and AuNRs1050 filters (Fig. 7b), reducing NIR range wavelengths from 730 to 1085 nm, hence predominantly acting as a notch filter. These basic examples of filter configurations aim to demonstrate the modularity of the filters and their potential for integration into existing systems that could benefit from the broad spectral range and simple fabrication process.

**Fig. 8:**
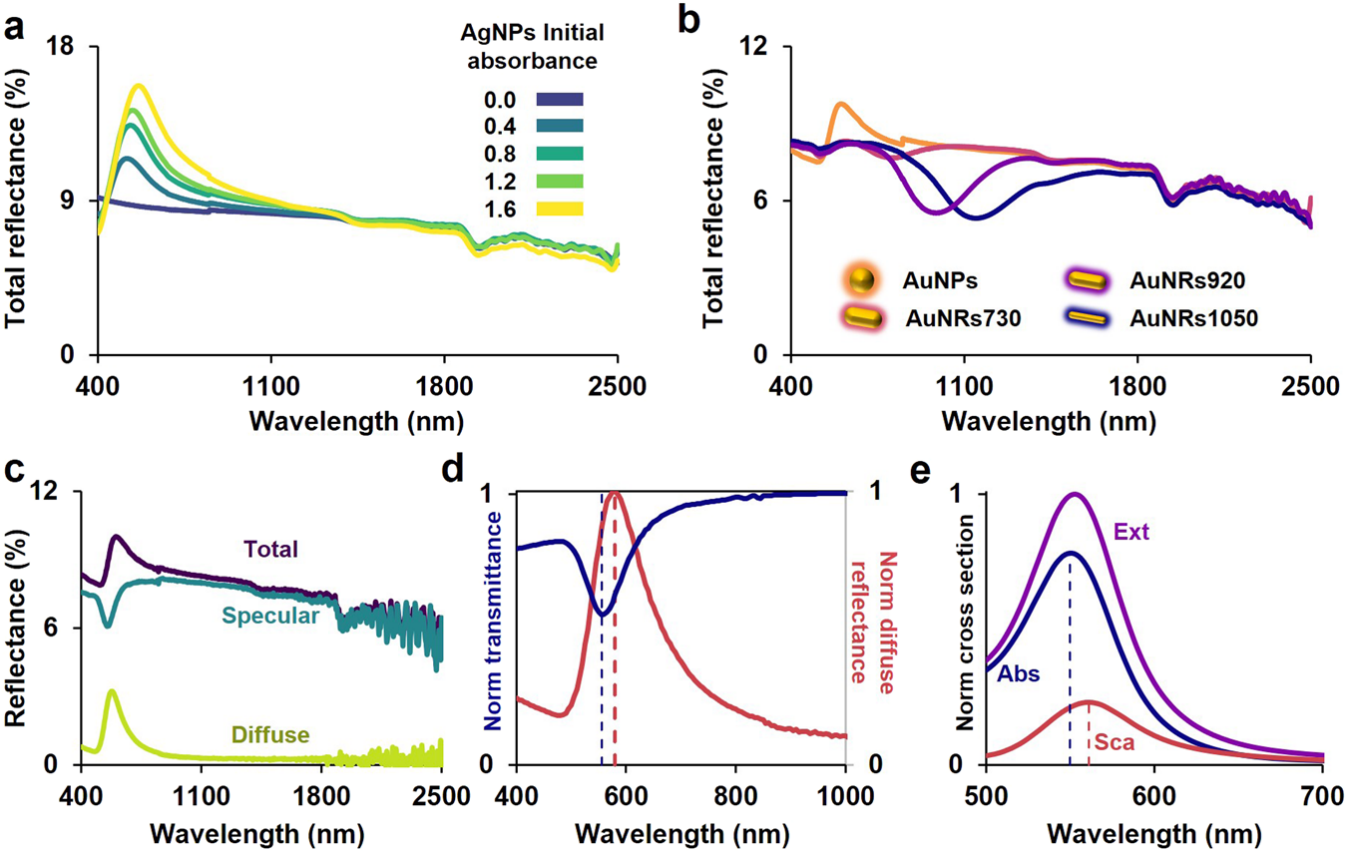
Reflectance of the gelatine light filters. **a** Increase the reflectance of the sample with the NPs concentration. **b** Tailored change in the reflectance in filters with the same level of transmittance. **c** Measured total and diffuse reflectance (ANPs), specular reflection is included as the difference between them. **d** Normalized transmittance and diffuse reflection of the light filters with AuNPs. **e** Calculated cross-sections for a 51 nm AuNP.

The presented gelatin-based filters primarily reduce incident light through absorption by the NPs. However, this method of light filtering is not always ideal. For example, IR filters used in buildings or automobiles to reduce energy consumption during summer prefer to reflect rather than absorb IR radiation^14^. This preference stems from the fact that a window absorbing IR radiation would act as a heater, despite preventing heat transfer by radiation. Therefore, we investigated whether the light not transmitted by our filters is entirely absorbed. For the filters with AgNPs, we measured the total reflectance over the different AgNPs concentrations (Fig. 8a). The increase in the total reflectance is directly correlated with the concentration of AgNPs. Interestingly, Fig. 8b shows that the reflected light can also be tailored (over the VIS-NIR range) with the constituent NPs. We are changing the ratio of reflectance to absorption at the same level of transmittance (see Fig. 6 for the transmittance). This result is likely due to the contribution of absorption and scattering to the total extinction of light. Hence, we measured the total and diffuse reflectance of the filter surface with AuNPs, and from these, we estimated the specular reflection, as shown in Fig. 8c. The total reflection accounted for an average of 7% of the untransmitted light (400–2500 nm), with the maximum in diffuse reflection redshifted from the maximum absorption. Plotting the normalized transmittance and diffuse reflection (Fig. 8d) highlights this shift (25 nm), which can be explained in terms of the calculated cross-sections of the far field. We employed the boundary element theory^15^ in the MNPBEM^16^ implementation in MATLAB to distinguish the absorption and scattering contributions to the total extinction (Fig. 8e), revealing an 11 nm redshift between them. This suggests that the dominant component of the diffusely reflected light can be attributed to scattering by the AuNPs. Although this contribution is minimal, it demonstrates that the NPs in the filters can manipulate incident light through absorption or scattering. This opens the possibility of creating filters that reflect light instead of absorbing it by changing the constituent NPs. An interesting material to explore could be silver nanodisks^17,18^.

## Conclusions

In this research, we have successfully pioneered the development and implementation of innovative gelatin-based light filters using metallic NPs, an endeavor inspired by the culinary utilization of biopolymers. This groundbreaking approach is expected to catalyze further research and exploration into the application of biomaterials across various sectors, including optics, materials science, electronics, and potentially beyond. Embracing the current environmental ethos, these optical filters are fashioned from biocompatible and eco-friendly materials, epitomizing the shift towards green and sustainable material research. The filters’ adaptability across the UV-Visible-NIR electromagnetic spectrum is a significant achievement, allowing for precise customization and functionality. Moreover, their modular nature facilitates easy integration into existing technological and scientific apparatus, enhancing their utility and applicability. The fabrication process of these filters is notably straightforward and cost-effective, eliminating the need for elaborate or high-cost equipment. This simplicity in production paves the way for widespread adoption and experimentation in both academic and industrial settings, potentially leading to novel applications and advancements in the field of material science and photonics.

## Methods

### Materials

Tetrachloroauric acid (HAuCl_4_·3H_2_O), hexadecyltrimethylammonium bromide 99% (CTAB), cetyltrimethylammonium chloride solution 0.78 M, sodium borohydride 99% (NaBH_4_), silver nitrate (AgNO_3_), hydrochloric acid 37% (HCl), Hydroquinone 99% (HQ), L-ascorbic acid 99% (AA) and tri-sodium citrate dihydrate 99.5% (Na_3_Cit) were purchased from Thermo Fisher Scientific. Milli-Q water (18.2 MΩ) was used in all preparations. Commercial gelatine was purchased from Neutral gelatine was purchased from Ewald-Gelatine.

### Characterization

TEM images were collected from a JEOL 1010 and 1011. Samples were prepared on carbon-Formvar-coated 200 mesh copper grids. SEM samples (carbon coated) were imaged with a JEOL JSM-6700f. UV-VIS-NIR spectroscopy was performed in a Jasco V-770 with the appropriated accessories: for transmittance (FLH-741) and for reflectance measurements (ISN-923). Optical images were taken with a regular smartphone under natural illumination in the laboratory. For the exposition for white light, the sample was left under a 50× during 1 h.

### Synthesis of AgNPs

AgNPs were synthesized using a modified version of a previously reported protocol^19^. Briefly, a AgNO_3_ solution (100 ml, 1 mM) was heated to boiling point. Then 2 ml of Na_3_Cit (2 ml, 34 mM) was rapidly injected into the boiling solution an let under vigorous stirring for 45 minutes. Finally, the solution was left to cool at room temperature.

### Synthesis of AuNPs with uniform diameters

Smooth AuNPs with 43 nm of diameter were synthesized by a modification of the seed-mediated growth protocol^20^. Firstly, AuNPs with 10 nm of diameter were grown by addition of 2 ml of HAuCl_4_ solution (5 × 10^−4^ M) using a syringe pump with injection rate of 2 ml/h in a solution containing 2 ml of CTAC 0.2 M, 1.5 ml of AA (0.1 M) and 50 μl of the initial seeds. After that, 2 ml of HAuCl_4_ solution (5 × 10^−4^ M) were injected (with injection rate of 2 ml/h) in a second growth solution containing 2 ml of CTAC 0.1 M, 13 μl of AA 0.1 M and 10 μl of the 10 nm seeds.

### Synthesis of AuNRs

AuNRs were prepared by the seed-mediated growth method following two protocols previously described^21,22^. In order to tune the longitudinal LSPR some modifications have been made in the addition of AgNO_3_ and seeds solutions in the growth process.

### Preparation of the initial, CTAB-Capped Au clusters (seeds)

A fresh aqueous NaBH_4_ solution (0.3 ml, 0.01 M) was rapidly added into a 4.7 ml of an aqueous solution containing HAuCl_4_ (2.5 × 10^−4^ M) and CTAB (0.1 M). The mixture was stirred a speed of 1000 rpm for 2 min, and then kept undisturbed at 27 °C for 3 h to ensure complete decomposition of the NaBH_4_.

### AuNRs with LSPR centered at 730 nm and 920 nm

AuNRs with LSPR centered at 730 nm and 920 nm, respectively, were synthesized by a modification of the seed-mediated growth protocol described by Scarabelli et al. ^2^. Briefly, 20 μl of the Au-seeds in the growth solution (for AuNR with LSPR at 730 nm) and 32 μl of the Au-seeds (for AuNR with LSPR at 920 nm) were added to a 10 ml grown solution with 120 μl of AgNO_3_ (0.01 M), and final concentration of HAuCl_4_ and CTAB, fixed at 5 × 10^−4^ M and 0.1 M, respectively.

### AuNRs with LSPR = 1050 nm

AuNRs with LSPR centered at 1050 nm were synthesized by a modification of the seed-mediated growth protocol described by Vigderman et all. ^22^. First, 0.5 ml of HQ (0.1 M) were added to a growth solution (10 ml) that contains 120 μl of AgNO_3_ (0.01 M) and final concentration of HAuCl_4_ and CTAB fixed at 5 × 10^−4^ M and 0.1 M, respectively. Next, we used 0.4 ml of seeds.

In the last step AuNPs and AuNRs were collected by centrifugation at 6000 rpm for 40 min, and then washed two times and redispersed in water for characterization.

### Plasmonic galatine-based light filters

The nanocomposites were obtained through the dispersion of plasmonic NPs on a gelatin matrix. To do this, a certain amount of gelatin was hydrated in cold water for 10 minutes and then dissolved in water at 35 °C. Then was mixed with colloidal NPs to obtain a solution with final concentration of 2% (w/v) gelatine and the desired NPs concentration (Eq. 1). After that, the plasmonic sol was transferred to petri dishes and kept at 4 °C for 48 h in a fridge. To dry the material, the petri dishes were left in an open atmosphere for 7 days. To detach the filters, they were pulled out with tweezers while deforming the mold.

### Numerical cross-section calculations

Using the MNPBEM^16^ tool box, we place a AuNP with diameter of 51 nm in an ideal dielectric media (*n* = 1.536)^8^. The NP was modeled as a “trisphere” object with 484 vertex. For the dielectric constant of gold we used experimental data previously reported^23^.

### Supplementary information

Supplementary Information

## Data Availability

The authors declare that all relevant data supporting the findings of this study are available within the paper.

## References

[1] Mortensen, A. et al. Re‐evaluation of xanthan gum (E 415) as a food additive. *EFSA J.* **15**. 10.2903/j.efsa.2017.4909 (2017). 10.2903/j.efsa.2017.4909 PMC7009887 32625570

[2] Mudgil D, Barak S, Khatkar BS (2014). Guar gum: processing, properties and food applications—a review. J. Food Sci. Technol..

[3] Habibi H, Khosravi-Darani K (2017). Effective variables on production and structure of xanthan gum and its food applications: a review. Biocatal. Agric. Biotechnol..

[4] Schrieber, R. & Gareis, H. Gelatine handbook: theory and industrial practice. (John Wiley & Sons, 2007).

[5] Stevens, P. In: Food stabilisers, thickeners and gelling agents (ed. A. Imeson) 116–144 (2009).

[6] Meiyazhagan A (2015). Electrically conducting nanobiocomposites using carbon nanotubes and collagen waste fibers. Mater. Chem. Phys..

[7] Cheirmadurai K, Biswas S, Murali R, Thanikaivelan P (2014). Green synthesis of copper nanoparticles and conducting nanobiocomposites using plant and animal sources. RSC Adv..

[8] Colusso E, Martucci A (2021). An overview of biopolymer-based nanocomposites for optics and electronics. J. Mater. Chem. C..

[9] Manocchi AK, Domachuk P, Omenetto FG, Yi H (2009). Facile fabrication of gelatin-based biopolymeric optical waveguides. Biotechnol. Bioeng..

[10] Chang H-H, Murphy CJ (2018). Mini gold nanorods with tunable plasmonic peaks beyond 1000 nm. Chem. Mater..

[11] Bastús NG, Merkoçi F, Piella J, Puntes V (2014). Synthesis of highly monodisperse citrate-stabilized silver nanoparticles of up to 200 nm: kinetic control and catalytic properties. Chem. Mater..

[12] Neupane MP (2011). Synthesis of gelatin-capped gold nanoparticles with variable gelatin concentration. J. Nanopart. Res..

[13] Zhang J-J, Gu M-M, Zheng T-T, Zhu J-J (2009). Synthesis of gelatin-stabilized gold nanoparticles and assembly of carboxylic single-walled carbon nanotubes/au composites for cytosensing and drug uptake. Anal. Chem..

[14] Butt, M. et al. Infrared reflective coatings for building and automobile glass windows for heat protection. **10342** OTT (SPIE, 2017).

[15] García De Abajo, F. J. & Howie, A. Retarded field calculation of electron energy loss in inhomogeneous dielectrics. *Phys. Rev. B* **65**, 10.1103/physrevb.65.115418 (2002).

[16] Hohenester U, Trügler A (2012). MNPBEM—a Matlab toolbox for the simulation of plasmonic nanoparticles. Comput. Phys. Commun..

[17] Naruse, M. et al. Randomness in highly reflective silver nanoparticles and their localized optical fields. *Sci. Rep.* **4**, 10.1038/srep06077 (2014). 10.1038/srep06077 PMC4133708 25123658

[18] Kiyoto N, Hakuta S, Tani T, Naya M, Kamada K (2013). Development of a near-infrared reflective film using disk-shaped silver nanoparticles. Fujifilm Res. Dev..

[19] Lee PC, Meisel D (1982). Adsorption and surface-enhanced Raman of dyes on silver and gold sols. J. Phys. Chem..

[20] Zheng Y, Zhong X, Li Z, Xia Y (2014). Successive, seed‐mediated growth for the synthesis of single‐crystal gold nanospheres with uniform diameters controlled in the range of 5–150 nm. Part. Part. Syst. Charact..

[21] Scarabelli L, Sánchez-Iglesias A, Pérez-Juste J, Liz-Marzán LM (2015). A “Tips and Tricks” practical guide to the synthesis of gold nanorods. J. Phys. Chem. Lett..

[22] Vigderman L, Zubarev ER (2013). High-yield synthesis of gold nanorods with longitudinal SPR peak greater than 1200 nm using hydroquinone as a reducing agent. Chem. Mater..

[23] Johnson PB, Christy RW (1972). Optical constants of the noble metals. Phys. Rev. B.

