# Supplementary material for: Eco-friendly and biocompatible gelatin plasmonic filters for UV-vis-NIR light
Source: Commun Chem. 2024 May 25;7:115. doi: 10.1038/s42004-024-01202-6 (PMC11128008; doi:10.1038/s42004-024-01202-6)
Supplement: Supplementary file 1 — Supplementary Information [file 42004_2024_1202_MOESM1_ESM.pdf]

Supplemental information for

Eco-friendly and Biocompatible Gelatin Plasmonic Filters for UV-Vis-NIR Light

I. Brian Becerril-Castro[1], Yoel Negrín-Montecelo[1], Josep Moreno[2], Miguel A. Correa-Duarte,[3]* Vincenzo Giannini,[4,5,6]* and Ramón A. Alvarez-Puebla[1,7]*

Supplementary Note 1. Formula 1 derivation

Assuming a linear relationship between the NPs' absorption and the optical path length (λ), and a volume for the liquid casted ($v_{ag+gelatin}$) in a standard petri dish as a mold (Φ = 9 cm).

The liquid (Ag + gelatin) will have an initial absorbance $Abs_{Ag+gelatin}$, that will change once the liquid adapts the shape of the mold Abs_{filter}. The relationship between them can be written as

$$Abs_{filter} = \frac{\lambda_{filter} * \ Abs_{Ag+gelatin}}{\lambda_{Ag+gelatin}}$$

The absorbance $Abs_{Ag+gelatin}$ will be determined by the amount of AgNPs (v_{np}) that are mixed with gelatin:

$$Abs_{Ag+gelatin} = \frac{\lambda_{Ag+gelatin} * Abs_{Ag} * v_{np}}{\lambda_{Ag} * v_{Ag+gelatin}}$$

Thus, we can look for the volume required as:

$$v_{np} = \frac{Abs_{filter} * \lambda_{Ag} * v_{Ag+gelatin}}{\lambda_{filter}}$$

The λ_{filter} can be expressed as:

$$\lambda_{filter} = \frac{v_{Ag+gelatin}}{\pi \left(\frac{\Phi}{2}\right)^2}$$

Finally, to measureλ_{Ag}we can use a cuvette with 1cm light path, to obtain the equation 1 in the text.

$$v_{np} = \pi \left(\frac{\Phi}{2}\right)^2 \frac{Abs_{filter}}{Abs_{np}} = \pi 4.5^2 \frac{log_{10}\left(\frac{100\%}{100\% - A(\%)}\right)}{Abs_{np}}$$

Supplemental Figures

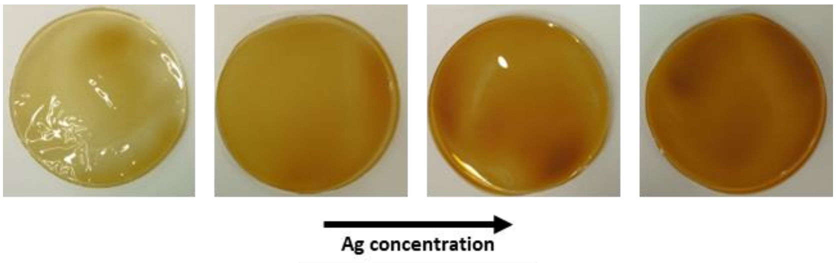

Supplementary Figure 1. Additional optical images of non-refrigerated materials with increasing Ag concentration. The formation of highly concentrated areas occurs during the trying process in all the tried concentrations (initial absorbance 0.4, 0.8, 1.2, and 1.6).

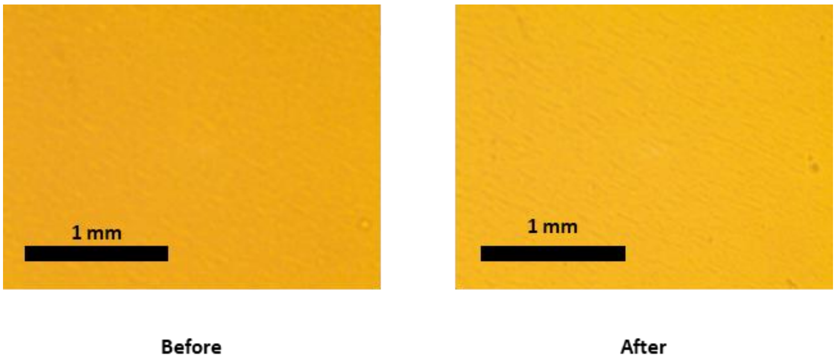

Supplementary Figure 2. Optical images of the films before and after exposition to white light during 1 h. Transmittance of the material remain unchanged.
